# Nerve Fiber Immunohistochemical Panel Discriminates between Nerve Sheath and Perivascular Wall Tumors

**DOI:** 10.3390/vetsci10010001

**Published:** 2022-12-21

**Authors:** Sílvia Sisó, Paola Marco-Salazar, Paola Roccabianca, Giancarlo Avallone, Robert J. Higgins, Verena K. Affolter

**Affiliations:** 1 Departments of Pathology, Microbiology and Immunology, School of Veterinary Medicine, University of California Davis, Davis, CA 95616, USA; 2 Pathology, Immunology Discovery, AbbVie, 100 Research Dr, Worcester, MA 01605, USA; 3 Department of Medicina i Cirurgia Animals, School of Veterinary Medicine, Universitat Autònoma de Barcelona, 08193 Bellaterra, Spain; 4 Department of Veterinary Medicine and Animal Sciences, University of Milan, 26900 Lodi, Italy; 5 Department of Veterinary Medical Sciences, University of Bologna, 40064 Ozzano dell’Emilia, Italy

**Keywords:** nerve sheath tumors, NST biomarkers, immunohistochemistry, laminin, Sox-10, periaxin-1, perivascular wall tumors, dog

## Abstract

**Simple Summary:**

Soft-tissue sarcomas are classified as nerve sheath tumors if originating within nerves. However, tumors of the nerves still pose a frequent challenge for diagnostic veterinary pathologists since identifying nerve histomorphology based only on the examination of hematoxylin-and-eosin-stained sections is often insufficient. In addition, there are no robust tumor-related biomarkers for a reliable diagnosis of nerve sheath tumors. Improving the anatomo-pathological diagnosis of nerve sheath tumors will contribute to a more accurate classification of soft-tissue sarcomas and their prognosis, ultimately aiding clinicians in making informed decisions for their patients. This current immunohistochemical study explores a combined panel of laminin, Sox-10 and periaxin-1 as an ancillary diagnostic approach to confirm the diagnosis of suspect nerve sheath tumors. The study demonstrates that this panel discriminates reliably between soft-tissue subtypes such as nerve sheath tumors and perivascular wall tumors.

**Abstract:**

Benign and malignant nerve sheath tumors (NST) pose a major challenge in routine diagnostic anatomic pathology because of shared histomorphological features with other soft-tissue tumors (STT). As a result, NST are often diagnosed as STT, a broad category that encompasses various entities including perivascular wall tumors (PWT) and that represents approximately 15% of all skin tumors in dogs. Immunohistochemistry (IHC) can assist the identification of histologic subtypes of STT. This IHC pilot study applies various markers largely expressed by peripheral nerves to twelve benign and six malignant NST and determines the intratumoral protein expression of laminin, periaxin-1, Sox-10 and S-100 in the NST subtypes. Furthermore, this study assesses the usefulness of peripheral nerve markers applied to diagnostic work cases and demonstrates the relevance of laminin expression patterns, periaxin-1 and Sox-10 in assisting the differentiation of NST from other STT, in particular from PWT.

## 1. Introduction

Soft-tissue tumors (STT) represent approximately 15% of all skin tumors in dogs [1] and comprise a diverse group of benign and malignant variants of nerve sheath tumors (NST) and perivascular wall tumors (PWT) amongst many other types [2]. While NST arise within nerves, PWT arise from the vascular wall. Benign NST (bNST) in domestic animals, similarly to their human counterparts [3,4,5], can be sub-classified into Schwannomas, perineuriomas or neurofibromas based on the prevalence of the specific nerve component that suffers neoplastic transformation (Schwann cells, perineurial cells or fibroblasts, respectively) [2,6,7,8,9]. Each bNST sub-type typically has morphological and ultrastructural features, including basal lamina of Schwann cells and pseudo-onion bulb formation of perineurial cells, which are crucial for a definitive diagnosis [7,8]. In contrast, malignant NST (mNST) are embodied in a single category due a general lack of distinctive growth patterns and cellular characteristics [2,7,8].

The proposed sub-classification of canine PWT comprises a mixed group of tumor sub-types that include hemangiopericytomas, angioleiomyosarcomas, angiofibromas, angioleiomyomas, angiomyofibroblastomas and myopericytomas [2,7]. Like mNST, some PWT sub-types, such as hemangiopericytomas, are rare and likely more aggressive [10].

At present, despite their distinctive cellular derivation, the microscopic distinction of STT into NST and PWT on routinely examined sections may be challenging in some cases because of several shared histological features. Transmission electron microscopy allows for confirmation of the tumor type but is not readily available or practical [11,12,13]. Immunohistochemistry assists in the definition of tumor cell origin by identification of the neoplastic cell phenotype. However, rarely one marker is informative enough because expression can be shared by different neoplasms, and thus a panel of antibodies is often required, such as, for example, laminin or collagen IV, which both can be expressed by NST and PWT [9,14]. For practical reasons, the goal of an optimal diagnostic panel would be to minimize costs while ensuring that the reduced number of markers is informative enough.

This retrospective study documents that a panel including anti-laminin, anti-Sox-10 and anti-periaxin-1 antibodies can assist in the confirmation of the diagnosis of NST and hence the differentiation from other STT, such as PWT.

## 2. Materials and Methods

### 2.1. Case Selection

In the first phase for marker optimization, a total of four NST, including one Schwannoma, one perineurioma, one neurofibroma and one mNST and one healthy peripheral nerve were evaluated. Those selected tumor samples contained both normal nerves and neoplastic growth, thus allowing for the robust assessment of Schwann cell IHC markers in components of residual healthy nerve fibers entrapped within the tumor as well as in tumor cells and/or stroma.

In the second phase the database at the William Pritschard School of Veterinary Medicine at UC Davis was searched for cases of NST. In addition, three PWT, fully characterized at the University of Milan, including an angioleiomyoma, a myopericytoma and an undifferentiated PWT, were evaluated for comparison.

In a third phase, the database at the William Pritschard School of Veterinary Medicine at UC Davis was searched for diagnosis of STT and the panel of Schwann cell IHC markers was evaluated in five STT to reach a definitive diagnosis.

### 2.2. Markers to Interrogate NST in Comparison to PWT

The selected antibody panel is detailed in Table 1. The panel targeted antibodies that recognize mostly nerve components, such as laminin (LAM), S-100, GFAP, Sox-10, periaxin-1 (PXN) and smooth muscle actin (SMA), but also proliferation markers including Ki-67 and phosphorylated histone H3 (PHH3) that could inform on the biologic behavior of neoplastic cells.

The markers were selected based on their expression within nerves and NST. Laminin is an important component of the basement membrane zone, and therefore is used in tumors of endothelial, smooth muscle, Schwann cell or perineurial cell origin [2,8]. Anti-S-100 and GFAP, recognizing Schwann cells, have been reported to be expressed by mNST [8,15]. Anti-periaxin-1 was included as a reliable marker for Schwann cell cytoplasm as it identifies the peripheral myelin sheath, and anti-Sox-10 was chosen as a nuclear marker of neural crest origin [8,16,17]. Smooth muscle actin (SMA) was included to detect vascular mural cells of PWT [2,16]. In addition, mitotic index (MI) was registered using anti-phosphorylated histone H3 (PHH3) and anti-Ki-67 (MIB-1 clone) labeling was used to document the proliferative index (PI).

### 2.3. Immunohistochemistry

Briefly, 4–5 µm thick sections were mounted on positive-charged glass slides and air-dried overnight at 37 °C. After deparaffinization and rehydration, the endogenous peroxidase activity was blocked with 0.3% hydrogen peroxide in methanol. Antigen retrieval procedures included either steam- or heat-induced epitope retrieval (HIER) with Target Retrieval Solution (DAKO, Carpinteria, CA, USA; S1699), or where appropriate, enzymatic digestion with Proteinase K (DAKO Carpinteria, CA, USA; S3020) following the manufacturer’s instructions. The antibody and blocking diluents and all the subsequent rinses were performed with PBS-Tween 20 (0.02%). Sections were blocked with 10% normal horse serum for 20 min. Then primary antibodies (Table 1) were applied for 1 h at room temperature. After rinsing, 4 + Detection System with anti-mouse link or anti-rabbit link and Streptavidin-HRP label (Biocare Medical, Pacheco, CA, USA; HM606, GR 608, respectively) was applied for all antibodies for 10 min with each reagent. Double rinsing was performed between each reagent application. Antigen detection was visualized using Vector NovaRed for peroxidase (SK-4800, Vector Laboratories, Newark, CA, USA), following the manufacturer’s instructions. Sections were counterstained with Mayer’s hematoxylin. Sections in which either diluent or normal serum substituted the respective primary antibody were included as negative controls. A brain lymphoma and a glioma tissue microarray were included as positive control tissue for GFAP, Ki-67 and PHH3. Peripheral nerve was the positive control tissue for all other markers.

### 2.4. Criteria for Evaluating the Immunoreactivity of Each Nerve Marker in NST

After testing the specificity of the nerve panel in nerve fibers, we qualitatively assessed the degree and extent of intratumoral immunoreactivity to each nerve fiber marker in all available NST. Evaluation of LAM, S-100, GFAP and SMA IHCs followed a binary read-out based on presence (positive) or lack (negative) of immunoreactivity in the whole tumor. For PXN, total counts of intratumoral nerve sheath profiles as well as cell immunoreactivity were evaluated in 10 HPF (one HPF equals a microscopic field at 40×, corresponding to 0.237 mm^2^) or whole tumor in small samples. Individual counts found in each HPF were recorded as a range of positive myelin profiles (denoted as X in Table 2) or cell counts (denoted as Y in Table 2). For Sox-10 positive cells, immunopositive nuclei were counted in 10HPF (2.37mm^2^) and again tabulated as a range of positive cells counted in one HPF. Immunoreactivity scores were interpreted as follows: low (0–5 Sox-10+ nuclei/HPF), intermediate (0–50 Sox-10+ nuclei/HPF) and high (above 50/HPF) scores of immunoreactivity. The PI or MI were generated as a percentage of immunoreactive cells to ki-67 and PHH3, respectively, from 2000 examined cells in tumor areas with the highest immunoreactivity.

## 3. Results

### 3.1. Intranerve NST

**Figure 1:**
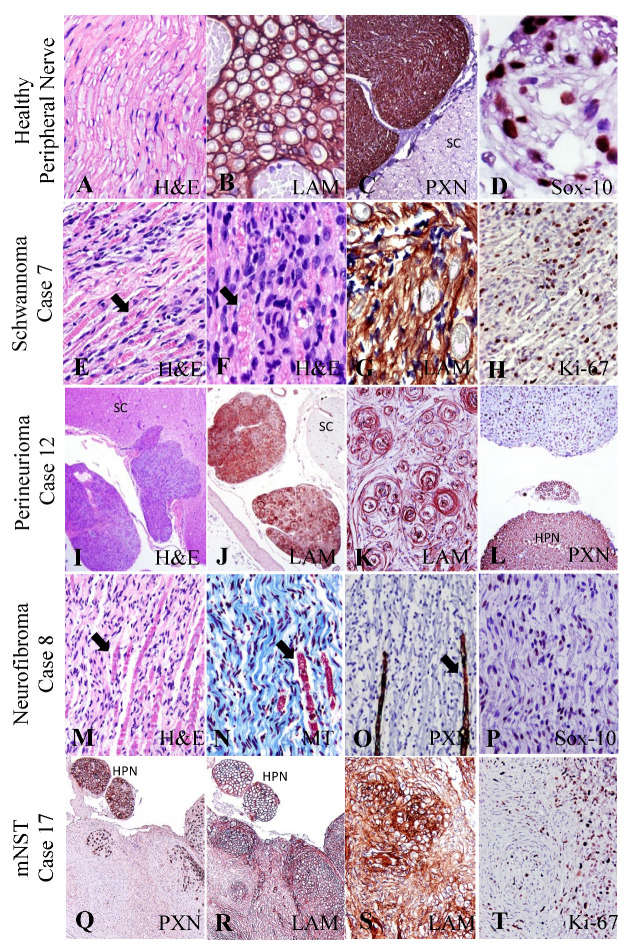
**Optimization of peripheral nerve markers in positive control tissue.** (**A**,**E**,**F**,**I**,**J**,**M**): The distinct morphology of a healthy nerve and the cells and tumor histogenesis of NST can be appreciated on H&E-stained tissue sections. (**B**,**R**) Laminin (LAM) immunoreactivity highlights the cytoplasmic Schwann cell membranes outlining the myelin sheath of healthy peripheral nerves (HPN). Confirmation by laminin (LAM) of basal lamina of cytoplasmic membranes in a Schwannoma (**G**), perineurioma (**J**,**K**) and mNST (**R**,**S**). Periaxin (PXN) detects peripheral myelin only. Note that the spinal cord (SC) is negative, while the spinal nerve root is strongly positive in healthy nerves (**C**) and in NST (**L**,**Q**). In the case of neurofibromas, Masson’s trichrome (MT) identifies the collagenous tumor stroma in blue (**N**) and PXN identifies nerve fibers entrapped within the tumor (**O**). (**E**,**M**–**O**) Arrows point at retained nerve fibers within NST. (**Q**–**S**) Neoplastic areas of a mNST have weak PXN and robust LAM immunoreactivity. Sox-10 directed to nuclei of Schwann cells identifies peripheral nerves (**D**) and neoplastic Schwann cells (**P**). Ki-67 immunolabels nuclei of neoplastic cells that are actively proliferating (**H**,**T**).

All markers included in the nerve panel immunoreacted with components of healthy nerve fibers or with intratumoral nerve fibers (internal positive controls) (Figure 1A–D). Laminin outlined nerve fibers (Figure 1B), PXN identified the myelin sheath (Figure 1C) and Sox-10 reliably recognized nuclei (Figure 1D). Similarly, neoplastic Schwann cells strongly immunoreacted to LAM in Schwannomas (Figure 1G), perineuriomas (Figure 1K), neurofibromas and mNST (Figure 1R,S).

In NST, LAM consistently outlined the peripheral membrane of individual neoplastic Schwann cells and perineurial cells as a continuum, as well as the perimeter of intratumoral blood vessels. The pattern of LAM recapitulated the fasciculate growth pattern of NST that was observed on H&E serially stained sections. PXN detected peripheral myelin and not myelin from nerve fibers of the CNS (Figure 1C). It strongly immunostained all nerve sheaths entrapped in NST (Figure 1L,O,Q). Occasionally, the cytoplasm of neoplastic Schwann cells can display focal, weak to strong, PXN immunopositive (Figure 1Q). Nuclear Sox-10 immunoreactivity, which was consistently visualized in healthy nerves (Figure 1D), was present in many neoplastic cells with a diffuse or patchy distribution in all NST tumors including neurofibromas (Figure 1P).

Other nerve panel markers such as S-100 and GFAP labeled the cytoskeleton of nerve fibers but were less robust markers if compared with LAM, PXN and Sox-10. S-100 was not specific to neoplastic cells since endothelial and inflammatory cells were weakly stained. GFAP inconsistently labeled nerve fiber components in healthy nerves, blood vessels and in NST. Last, SMA positivity was observed variably in the epineurium and perineurium, the tumor pseudocapsule and the surrounding blood vessels.

### 3.2. Comparison of Immunoreactivity of NST and PWT

**Figure 2:**
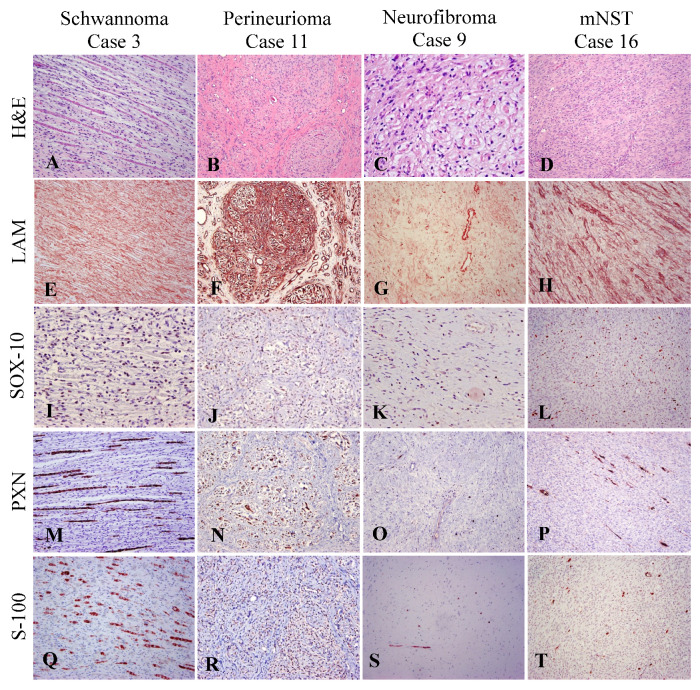
**Peripheral nerve IHC panel phenotype benign and malignant peripheral nerve sheath tumors (NST).** The nerve markers to be tested in NST included laminin (LAM), Sox-10, periaxin-1 (PXN) and S-100. (**A**–**D**) H&E of each NST sub-type is given for histomorphological context. In all NST, LAM provided robust results by variably highlighting the cytoplasmic membrane of Schwann cells and of perineurial cells (**E**–**H**). Sox-10 was consistent in identifying nuclei from neoplastic Schwann cells and those entrapped within nerve fibers. (**I**,**J**) Schwannoma and pe- rineurioma tumors had the highest scores for Sox-10, whereas neurofibromas (**K**) and mNST (**L**) show intermediate and low scores. (**M**–**T**) PXN, like S-100, strongly labeled fragmented myelinated fibers entrapped within tumors. In perineuriomas, they labeled myelin surrounding intact axons.

This study includes a total of 18 tumors diagnosed as NST based on morphological features. The 18 NST included 12 bNST, previously diagnosed as Schwannomas (7/12), neurofibromas (2/12) and perineuriomas (3/12) and 6 mNST. Results of the nerve IHC panel applied to 12 bNST and 6 mNST are summarized in Table 2. Laminin, Sox-10 and PXN were tested in 18/18 NST, S-100 in 14/18 and GFAP in 15/18 cases due to limited tissue availability. Reactivity in bNST was as follows: 12/12 (100%) with LAM, 11/12 (92%) with PXN, 10/12 (83%) with Sox-10, 8/9 (89%) with S-100 and, 5/9 (55%) with GFAP. In mNST LAM, Sox-10 and PXN were positive in all 6 (100%) of the cases. Positivity for other markers, GFAP (4/6; 67%) and S-100 (4/5; 80%), was lower. Figure 2 illustrates the immunostaining pattern of LAM, Sox-10, PXN and S-100 in a representative NST. In all NST, LAM displayed variable positive intensity across the tumor but a consistent linear pattern outlining the cytoplasm of neoplastic cells and occasional entrapped nerve fibers (Figure 2E–H). Similarly, Sox-10+ counts varied between tumors and within a tumor. Benign NST showed a range of scores. In 4/12 cases (Table 2), Sox-10 protein expression was low with high variation among fields of view (FOV) as several FOVs had a 0 score of Sox-10-positive cells and the highest count in a single FOV was five positive cells. Instead, an FOV from a high expressor (3/12) such as bNST cases 3, 6, and 7 had Sox-10 protein expression in most neoplastic cells. All malignant NST showed low to intermediate Sox-10 scores. Cases 5 and 10 had high tissue background staining with Sox-10 and cells exhibited cytoplasmic rather than nuclear staining; these cases were not considered positive for Sox-10. Examples of a high (Case 3), intermediate (Cases 11 and 16) and low expression (Case 9) are illustrated in Figure 2I–L. For PXN, all 18 NST except two Schwannomas (Cases 2 and 5) had intratumoral PXN positive nerve fibers. The quantity of PXN+ nerve fibers varied across tumors. In general, tumors contained multiple short fragments of nerve fibers with myelin expressing PXN. Numbers of PXN-positive myelin profiles varied markedly between FOVs, ranging from 0 to 100. The range of PXN+ myelin profiles counted in a HPF are displayed in Table 2 and in Figure 2M–P. In addition, in a few NST (5/18; 28%), the cytoplasmic processes of neoplastic cells immunoreacted with PXN (Cases 1, 2, 4, 9 and 13). Additional primary antibodies included widely used markers like S-100 and GFAP, that were included for comparative purposes. Like PXN+ myelin profiles, S-100 immunoreacted with residual fragmented nerve fibers enclosed in the tumor (Figure 2Q–T) and was able to detect the majority of NST (12/14; 86%). S-100 marker was expressed by 89% of benign NST and 80% of mNST. Assessment of intracellular immunoreactivity to S-100 was challenging because in many cases it was difficult to interpret or was regarded as inconclusive due to high background. GFAP immunoreacted to parts of nerve fibers in 60% of the NST cases (9/15).

**Figure 3:**
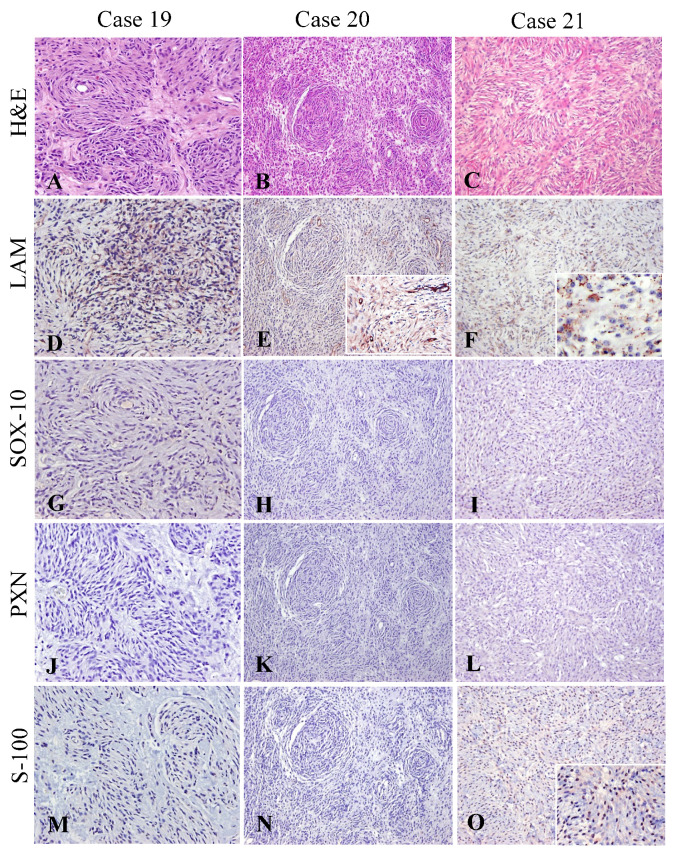
**Peripheral nerve IHC panel phenotype of perivascular wall tumors (PWT).** The nerve markers to be tested in PWT included laminin (LAM), Sox-10, periaxin-1 (PXN) and S-100. (**A**–**C**) H&E of each NST sub-type is given for histomorphological context. (**D**–**F**) All PWT display patchy areas of LAM immunoreactivity. Laminin variably decorated the cytoplasm of some neoplastic cells in a discontinuous stippled dotting pattern (insets in **E** and **F**). None of the PWT immunoreacted to Sox-10 (**G**–**I**) or PXN (**J**–**L**). S-100 immunopositivity was not detected in two PWT cases (**M**,**N**). In the third PWT case, Case 21, S-100 decorated the cytoplasm of some cells (**O**, inset).

**Figure 4:**
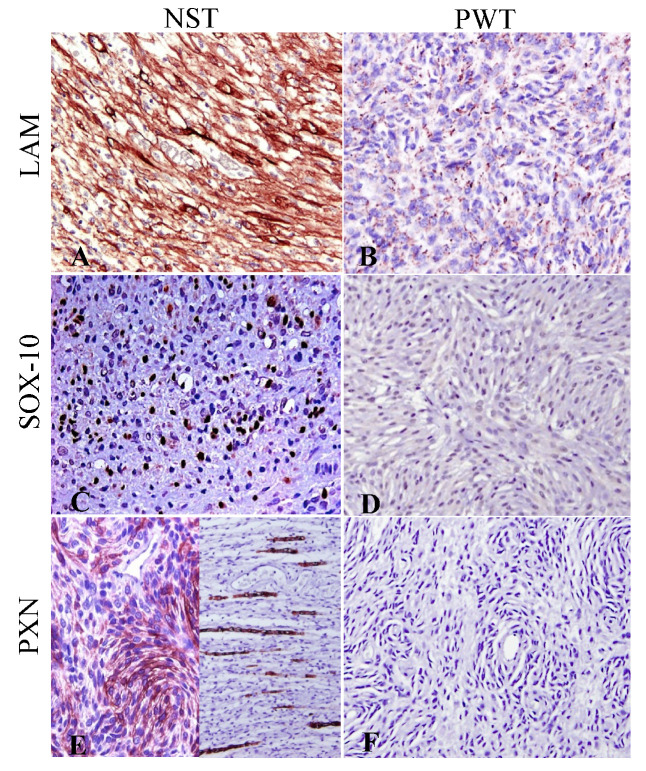
**Discriminatory IHC panel between NST and PWT.** The selected PNS panel including laminin (LAM), Sox-10 and periaxin-1 (PXN) was compared between NST and PWT. Laminin was detected in all tumors but the LAM immunolabeling pattern of PWT was different than in NST (**A**,**B**). NST retained Sox-10 and PXN immunoreactivity, whereas PWT did not (**C**,**D**). Note that PXN immunoreactivity can be observed in the cytoplasm of neoplastic cells (**E**, left) and/or in nerve sheaths retained within the tumor (**E**, right) in NST, but no PXN was detected in PWT (**E**,**F**).

The expression of LAM, Sox-10 and PXN was investigated in PWT (Figure 3 and Table 2). LAM expression was observed in all three PWT, with a patchy distribution and a characteristically granular/punctate expression pattern in the cytoplasm of neoplastic cells (Figure 3D–F). No PWT immunoreacted with Sox-10 and PXN (Figure 3G–L). On the contrary, S-100 marker was expressed in one-third of the PWT (Figure 3M–O). SMA was not detected in NST neoplastic cells but was present in two-thirds of PWT. Expression of LAM, Sox-10 and PXN in NST and PWT is illustrated and compared in Figure 4.

All NST tumors, except one neurofibroma (Case 9), displayed similar indices or percentages of Ki-67+ proliferating cells and of PHH3+ mitotic cells (Table 2). Immunohistochemistry for PHH3 resulted in a strong and sharp immunolabeling and detected nuclei with no mitotic morphology on H&E. Overall, the intragroup averaged PI and MI were highest in mNST (30 and 24, respectively) compared to benign NST (10 and 16, respectively) and to PWT (2 and 1, respectively). Neurofibromas and PWT had the lowest PI and MI.

### 3.3. Routine Laboratorial IHC Approach to the Diagnosis of NST: 4 Cases

**Figure 5:**
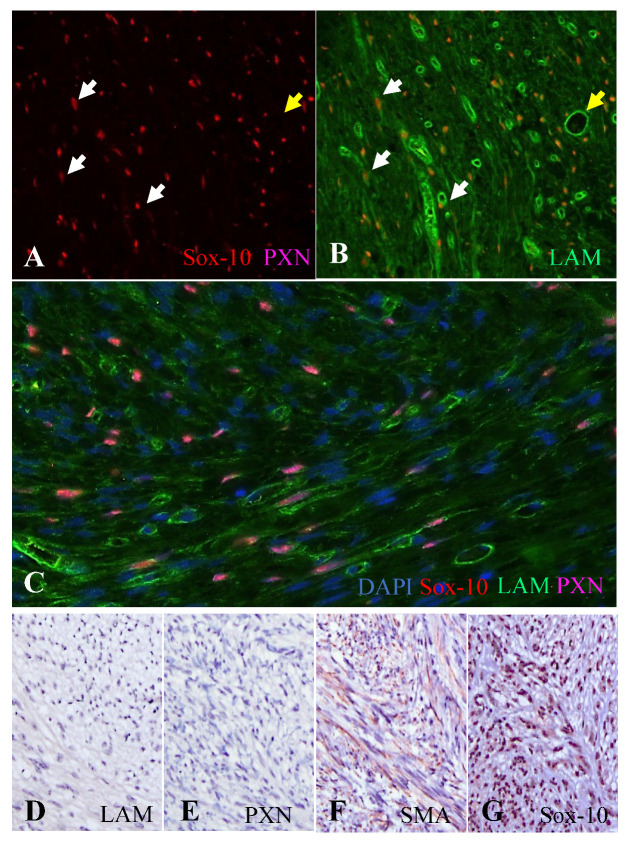
**Examples of diagnostic IHC work in STT.** Cases 25 (**A**–**C**) and 26 (**D**–**G**). A multiplex immunoflurorescence IHC for laminin (LAM), Sox-10 and periaxin-1 (PXN) on a benign NST (**A**–**C**) demonstrates frequent Sox-10+ nuclei (in red) of Schwann cells with no detected PXN+ axons (**A**). Note that LAM+ myelin sheaths (in green) with Schwann cells in red (white arrows) can be distinguished from blood vessels (yellow arrow) in the merge image with LAM on (**B**). (**C**) Merge immunofluorecent IHC image confirming that approximately 40% of nuclei are Sox-10+ (magenta) due to the superimposition of DAPI (in blue) and Sox-10 (in red). A tentative diagnosis of PWT (**D**–**G**) was rejected based on a negative result to LAM and PXN with strong immunostaining for SMA and Sox-10.

Five cases listed as STT in the database of the William Pritschard School of Veterinary Medicine, UC Davis, were evaluated with the abovementioned panel. Detailed results are listed in Table 3. Examples of the IHC panel for two cases are shown in Figure 5.

## 4. Discussion

This pilot study demonstrated that the combined evaluation of LAM, Sox-10 and PXN in the current NST and PWT case series helped differentiate between these two entities and has a diagnostic value in the specific identification of NST. In addition, immunohistochemical detection of these cell-specific antigens proved effective in accurately differentiating NST from PWT.

Previous studies have similarly focused on the differential diagnosis between NST and PWT using immunohistochemical panels [16,18,19]. Our study agrees with these published data by confirming that NST, different than PWT, are not immunoreactive to muscle markers such as SMA. In terms of NST immunohistochemical diagnosis, laminin (LAM) has been described as a useful marker [8,12,20] but has not been regularly included in the panel for NST diagnosis. As previously noted, S-100 and LAM are not exclusively expressed in NST, but one important result of this current study is that LAM was consistently positive in NST and that its distinct staining pattern assisted in its differentiation from PWT. Ideally, prospective studies on NST and PWT case series should include LAM to confirm the consistency of our result.

The selection of neural markers targeting Schwann cells, perineurial cells and neural crest derived cells commonly includes S-100, Claudin-1, PXN and NGFR, which have been variably observed in mNST [19]. However, the selection of the best individual markers continues to be a difficult task because each single marker used alone is not specific enough, further stressing the necessity of a better panel and possibly a multistep approach in conditions of economic constraints. For example, S-100 and Claudin-1 might not be the first-choice markers because they are expressed in a highly variable and heterogeneous fashion and do not seem specific enough to diagnose mNST since they can be expressed also in PWT [16]. A recent comprehensive study assessed Sox-10, Claudin-1, CNPase and GFAP in 79 canine NST [15]. The authors observed that, except for CNPase, the combination of markers detected more than 90% of bNST, concluding that 67% and 71% of mNST had retained Sox-10 and Claudin-1 immunoreactivity, respectively. Our data parallel the observations that GFAP, which is expressed in a subset of myoepithelial cells [2] and Schwann cells [15], was less expressed in this case load compared to other markers and was dramatically reduced in mNST compared to bNST [15]. Therefore, our results agree with these previous observations, adding that Sox-10 immunoreactivity was more sensitive than GFAP’s for mNST diagnosis. Additionally, and in contrast to their results [15], we did not observe decreased Sox-10 expression in mNST when compared to bNST. Based on our findings, GFAP seemed to co-localize with S-100 in serial sections but detected fewer numbers of myelinated fibers.

Additionally, S-100 and PXN performed similarly, overall detecting 86% and 94%, respectively, of all examined NST. While CNPase was negative in NST [15], PXN was expressed by most NST, highlighting it as a useful marker to consider. The recent literature regarding people and Tasmanian devils further supports the usefulness of neural-crest-cell-associated-markers such as Sox-10 and PXN for a more reliable diagnosis of NST [5,21]. In a small fraction of our NST, PXN was focally present in the cytoplasm of neoplastic Schwann cells, a finding already described in a cat Schwannoma [22]. Supporting our findings, the human WHO classification recommends the use of S-100 and Sox-10 as Schwann cell markers that can aid the diagnosis of NST [5]. However, confirming further observations made for human STS [23], in our study Sox-10 represented a more robust and overall better marker than S-100 for NST diagnosis based on its distinct nuclear labeling, a low background, and the more consistent immunostaining reproducibility. Moreover, because S-100 is expressed not only by Schwann cells but also by chondrocytes, adipocytes, and melanocytes [2] it is not specific as previously believed. Confirming this observation, S-100 was expressed in one PWT of this case series. Moreover, Sox-10 should be considered a better marker for Schwann cells than GFAP when detecting malignant NST due to a decreased expression of GFAP. Importantly, Sox-10 and PXN were useful for the discrimination between NST and PWT since Sox-10 and PXN detected 83% and 92% of benign NST, respectively, and 100% of mNST (Table 2) while PWT were negative. Taking our IHC data, we can conclude that LAM, Sox-10 and PXN used in combination improved the accuracy of NST diagnosis and assisted in their distinction from PWT. However, we believe that these results should be confirmed by assessing a larger number of PWT and a larger caseload of STT in general.

Despite their usefulness, there are some considerations regarding PXN and Sox-10 expression in NST that should be made. Tumor distribution of PXN and Sox-10 were variable across the fields of view (FOV) within the same tumor and between normal nerve fibers. Sox-10 immunoreactivity in one FOV could be 0 and as high as 50 positive cells in the adjacent FOV. As a result, evaluating the whole tumor sample or at least 2.37 mm^2^ of tumor is critical when assessing PXN and Sox-10 reactivity. More so, examination of a small biopsy sample could equivocally report a false negative. For PXN, it is important to assess the presence of myelin sheath profiles from healthy nerve fiber entrapped within the tumor as well as the presence of positive cells. Almost all examined NST had entrapped PXN+ myelin profiles and in addition, 5 out of 20 NST displayed the cellular PXN immunostaining pattern. A single case was negative for both patterns of PXN immunolabeling, which represented 5% of the NST caseload. Our results contrast with the study of Suzuki et al. [16], where PXN protein expression was scarcely detected in 10% of mNST. As for Sox-10, sample size can limit the effectiveness of PXN to detect NST. Additionally, it remains to be determined if sample processing protocols or autolysis may affect the presence of antigenic PXN epitopes and its subsequent detection. An alternative marker highly expressed by nerves, PNP9.5, has been reported to be expressed in 90% of canine NST [20], and might represent a second option in those NST cases where PXN is not expressed.

Thoughtful consideration of which markers need to be included during the study design, ensuring optimization of IHC and quality of samples, and providing a careful examination with standardized evaluation metrics are a few of the pre-requisites for driving progress in the challenging diagnostic field of STT. Our approach using LAM, Sox-10 and PXN with the conventional marker SMA was useful in diagnosing NST. Specifically, LAM and Sox-10 are expressed by different cell types. Laminin detects basement membranes present in endothelial cells, smooth muscle cells and Schwann cells and their corresponding tumors [2]. Just to complicate it further, despite Sox-10 being a good marker for tumors that originate from neural-crest-derived cells [24], myoepithelial cells and alveolar rhabdomyosarcoma can also have Sox-10 expression [25]. Moreover, neural crest tumors of melanocytic or nerve sheath origin may express Sox-10, alpha-SMA and S-100 [25,26]. Thus, it remains unknown if Sox-10 and PXN are expressed in other non-PWT STT, and this should be addressed in further studies to confirm or contrast their role in specifically identify NST. Unfortunately, the usefulness of NGFR was not assessed in the current study, but it has been suggested also as a potential marker to distinguish mNST from PWT and may represent a good addition to a panel for the differentiation of STT [16].

An example of a complex case was Case 26, where a definitive diagnosis was not achieved. For this case, a NST diagnosis was excluded based on lack of LAM and PXN; however, the tumor expressed Sox-10 and SMA. SMA, which was consistently negative in our NST caseload, is expressed in myofilaments of smooth muscle cells, myofibroblasts and pericytes and has been reported in PWT, leiomyosarcomas, chondrogenic tumors, rhabdomyosarcomas, melanomas, basal cell carcinomas and other tumor types [2,26]. However, one of our examined PWT had no SMA protein expression. Cautiously selecting more than one conventional marker expressed by tumor types and their cell counterparts [2] might be a good practice. For example, we could have selected a conventional skeletal muscle protein instead of, or in addition to, SMA in Case 26 to confirm a rhabdomyosarcoma or Sox-9 to confirm chondrocytes as the cell of origin [25,27].

Following resection, local recurrence rates in STSs are low in general but vary according to histologic grade and completeness of surgical margins [28]. It seems then appropriate that prospective studies on STT should include markers that could inform on tumor biological behavior. Since high mitotic index is prognostic for reduced survival time in STS, mNST, which had the highest PI and MI scores should bear a worse prognosis. PHH3 was an objective and sensitive method to detect mitotic figures in our STT subpopulations. Additionally, MI were higher than PI in a few cases. The potential unreliability of Ki-67 IHC readouts [29] and the suitability of PHH3 as an ancillary marker have been addressed in oncology [30,31,32]. Moreover, PHH3 expression in human astrocytomas and meningiomas have provided a relevant, simple, and exact method to accurately grade such tumors [30,31]. One would hope that future veterinary studies that integrate objective and more accurate quantitative measures to improve morphological subjective scoring systems in STT will help standardize their grading and make better informed decisions.

## 5. Conclusions

Canine soft-tissue tumors (STT) represent a broad category of tumors that can arise from various tissue components, including peripheral nerves for NST or various components of blood vessel walls for PWT. Morphologically these tumors may share similar growth patterns such as arrangement in short fascicles, whorls, or storiform areas composed of spindle to fusiform to plump cells with variable amounts of a fibrillar collagenous stroma. When specific patterns and tissue components are not visible their distinction may be challenging, and for this reason, the usefulness of their differentiation, despite its importance, has not been investigated yet. Moreover, currently available routine immunohistochemical panels of antibodies for differentiating these two tumor groups in dogs are very limited and often not specific enough. For all the above listed reasons, despite the cell of origin tends to dictate the behavior of a particular tumor type, most STS are classified and graded according to common histological features, mitotic index, and completeness of surgical margins rather than by their histogenetic origin. This deficiency has generated much diagnostic confusion in the veterinary literature regarding their histological classification. Such confusion also has major implications for an accurate prognosis and therefore limiting clinicians in making informed decisions for their patients. This current study favors a combined panel of LAM and Sox-10 and possibly PXN, for improving the diagnosis of STT of suspect neural origin, aiding in the proper diagnosis and grading of NST.

Because of this pilot IHC study, we suggest the introduction of LAM, periaxin-1 and Sox-10 in the diagnostic IHC to identify NST. If costs are a factor to consider, it is recommended to use LAM and Sox-10 as a first, paired IHC panel; PXN and SMA as a second step may be used in conjunction with NGFR and PNP9.5. Future studies that investigate expression of these markers in a larger number of STT and also including other tumor types will help confirm our findings and aid the molecular diagnosis of STT. 

## Figures and Tables

**Table 1:** Detailed information of antibodies included in the study.

Primary Antibody (Abbreviation)	Provider (Catalog Number)	Pre- Treatment	Primary Antibody Dilution	Targeted Antigen
Glial fibrillary acidic protein (GFAP)	Dako (Z0334)	Proteinase K, RT, 5 min.	1:600	Nerve fiber skeleton
Laminin (LAM)	BioGenex (PU078-UP)	0.2% protease, 37 °C, 10 min.	1:40	Basement membrane
Periaxin-1 (PXN)	Sigma-Aldrich (57716)	HIER ^1^	1:1000	Peripheral myelin
Sox-10	Santa Cruz (SC-17342)	HIER	1:100	Neural crest cells
S-100	Vector (VP-S276)	HIER	1:600	Neural crest cells /myelin
Smooth muscle actin (SMA)	BioGenex (MU128-UC)	SIER ^2^	1:300	Smooth muscle cells
Marker of proliferation (Ki-67)	Dako (M7240)	HIER	1:40	Proliferating cells
Phosphorylated histone H3 (PHH3)	Chemicon (06-570)	HIER	1:4000	Mitotic cells

RT, room temperature; min., minutes; ^1^ Heat-induced epitope retrieval with citrate pH 6.1, 95 °C, 30 min; ^2^ Steam-induced epitope retrieval with EDTA pH 9, RT, cool down for 20 min.

**Table 2:** Immunohistochemical assessment of NST and PWT.

STT	Age (Years)	Anatomical Location	H&E Dx	GFAP	LAM	PI	MI	PXN	Sox-10	S-100	SMA
1	5	Trigeminal n.	Schwannoma	Neg	Linear	20	20	<25/<5	5–50	Pos	Neg
2	6	C5-C6 spinal n. roots	Schwannoma	Neg	Linear	25	60	0/<1000	0–20	Pos	Neg+
3	6	L5-S1 n. roots	Schwannoma	Pos	Linear	1	3	<200/0	>500	Pos	Neg
4	8	L1-L2 spinal n. root	Schwannoma	Neg	Linear	15	10	<100/<100	0–5	Pos	Neg
5	3	C1-C2 spinal n. root	Schwannoma	Neg	Linear	5	20	0/0	Inconclusive	Neg	Neg
6	8	Trigeminal n.	Schwannoma	n/a	Linear	n/a	n/a	0-20/0	>500	n/a	n/a
7	12	Radial n.	Schwannoma	n/a	Linear	30	n/a	200/0	50–200	n/a	Neg
				**1/5**	**7/7**	**16**	**23**	**6/7**	**6/7**	**4/5**	**0/6**
8	3	Shoulder	Neurofibroma	Pos	Linear	1	0	0–20/0	0–5	Pos	Neg+
9	9	Cranial n.	Neurofibroma	Pos	Linear	1	20	3–100/<5	0–5	Pos	Neg
				**2/2**	**2/2**	**1**	**10**	**2/2**	**2/2**	**2/2**	**0/2**
10	not known	Cervical n. root	Perineurioma	Pos	Linear	20	20	0–100/0	Inconclusive	Pos	Neg +
11	9	C2 spinal n. root	Perineurioma	Pos	Linear	5	10	100/0	5-50	Pos	Neg
12	5	Cervical n. root	Perineurioma	n/a	Linear	n/a	n/a	100/0	0-20	n/a	n/a
				**2/2**	**3/3**	**13**	**15**	**3/3**	**2/3**	**2/2**	**0/2**
			**Benign NST**	**5/9**	**12/12**	**10**	**16**	**11/12**	**10/12**	**8/9**	**0/10**
13	4	L brachial plexus	mNST	Pos	Linear	30	50	0–200/50	0–20	Neg	Neg
14	4	Cauda equina	mNST	Pos	Linear	25	4	0–100/0	5–50	Pos	Neg
15	10	L brachial plexus	mNST	Pos	Linear	25	15	5–50/0	0–20	Pos	Neg+
16	12	L brachial plexus	mNST	Pos	Linear	40	10	0–100/0	5–50	Pos	Neg+
17	7	L4 spinal	mNST	Neg	Linear	30	60	0–100/100	0–5	Pos	Neg
18	4	Suprascap n.	mNST	Neg	Linear	n/a	7	0–100/0	5–50	n/a	Neg
			**Malignant NST**	**4/6**	**6/6**	**30**	**24**	**6/6**	**6/6**	**4/5**	**0/6**
			**NSTs**	**9/15**	**18/18**	**20**	**20**	**17/18**	**16/18**	**12/14**	**0/16**
19	9	Antebrachium	PWT	n/a	Punctate	1	0	0/0	0	Neg	Neg
20	12	Scrotum	PWT	n/a	Punctate	0	3	0/0	0	Neg	Pos
21	11	Scapula	PWT	n/a	Punctate	5	0	0/0	0	Pos	Pos
			**PWTs**	**n/a**	**3/3**	**1**	**2**	**0/3**	**0/3**	**1/3**	**2/3**

n., nerve; spinal, intramedullary; Neg+ denotes cases that show SMA immunoreactivity in the pseudocapsule of the tumor. PI (proliferative index) and MI (mitotic index) denote percentages of ki-67^+^ proliferative cells and PHH3^+^ mitotic figures, respectively. For PXN (X/Y), X denotes counts of positive myelin sheath profiles in one HPF, and Y denotes counts of positive cells in 1HPF. For Sox-10, a range of positive nuclei counts is given per one HPF.

**Table 3:** Evaluation of five STT with antibody panel to achieve a more specific diagnosis.

Case	Age (Years)	Tumor Location	Previous Diagnosis	LAM	Sox-10	PXN	SMA	Others	Final Diagnosis
22	12	Oral Mass Maxilla	STT Grade III	80% linear positivity	100% positive	Neg.	Neg	Desmin neg. PNL-2 neg.	mNST
23	1	Pelvic limb	STT Grade II	Punct Cyto	Neg.	Neg.	Nd	Nd	PWT
24	9	Pelvic limb	STT Grade II	Punct Cyto	Neg.	<5 MSh	Nd	Nd	PWT
25	5	Cervical area	STT Grade I	Linear positivity	20–200 counts positive	Nd	Nd	Nd	bNST
26	7	Rib, vertebra	STT; PWT?	Neg.	100% positive	Neg.	Neg.	Desmin neg.	neural crest tumor?

Punct Cyto, minimal punctate/stippled cytoplasmic; MSh, myelin sheath; Nd, not done.

## Data Availability

Data is contained within the article. There is no Supplementary Material.
